# Effects of mixed provider payment systems and aligned cost sharing practices on expenditure growth management, efficiency, and equity: a structured review of the literature

**DOI:** 10.1186/s12913-018-3779-1

**Published:** 2018-12-27

**Authors:** Isabelle Feldhaus, Inke Mathauer

**Affiliations:** 1 Department of Global Health and Population, Harvard T.H. Chan School of Public Health, 665 Huntington Avenue, Boston, MA 02115 USA; 2 Department of Health Systems Governance and Financing, World Health Organisation, Avenue Appia, 1211 Geneva, Switzerland

**Keywords:** Provider payment, Strategic purchasing, Blended payment, Bundled payment, Integrated care

## Abstract

**Background:**

Strategic purchasing of health care services has become a key policy measure on the path to achieving universal health coverage. National provider payment systems for health services are typically characterized by mixes of provider payment methods with each method associated with distinct incentives for provider behaviours. Reaching incentive alignment across methods is critical to enhancing the effectiveness of strategic purchasing.

**Methods:**

A structured literature review was conducted to synthesize the evidence on how purposively aligned mixed provider payment systems affect health expenditure growth management, efficiency, and equity in access to services with a particular focus on coordinated and/or integrated care management.

**Results:**

The majority of the 37 reviewed articles focused on high-income countries with 74% from the US. Four categories of payment mixes were examined in this review: blended payment, bundled payment, cost-containment reward models, and aligned cost sharing mechanisms. Blended payment models generally reported moderate to no substantive reductions in expenditure growth, but increases in health system efficiency. Bundled payment schemes consistently report increases in efficiency and corresponding cost savings. Cost-containment rewards generated cost savings that can contribute to effective management of health expenditure growth. Evidence on aligned cost-sharing is scarce.

**Conclusion:**

There is lacking evidence on when and how mixed provider payment systems and cost sharing practices align towards achieving goals. A guiding framework for how to study and evaluate mixed provider payment systems across contexts is warranted. Future research should consider a conceptual framework explicitly acknowledging the complex nature of mixed provider payment systems.

**Electronic supplementary material:**

The online version of this article (10.1186/s12913-018-3779-1) contains supplementary material, which is available to authorized users.

## Introduction

As one of the generic sub-functions of health financing, purchasing involves the allocation of resources to health service providers [1]. In efforts to devise more efficient approaches to purchasing services, countries have moved away from *passive* purchasing (i.e., no selection of providers, no performance monitoring, and/or no effort to influence prices, quantity, or quality of care) to engage in *strategic* purchasing [2]. Strategic purchasing involves linking provider payment to information, such as provider performance or population health needs, to align funding and incentives to achieve improved efficiency, accountability, service delivery, and equity [3, 4]. On the path to universal health coverage (UHC) amidst an increasing burden of chronic disease, this is considered a key policy measure and requires policy analysis to identify what effective strategic purchasing means in a particular context.

Provider payment is a central element of purchasing. Paying providers is a complex process with most countries applying a mix of provider payment methods (PPMs), i.e. there are at least two payment methods in place, such as budget allocations, fee-for-service (FFS), salary, capitation, or value-based payment [3, 4]. For example, an individual provider might receive budget allocations from one purchaser and FFS from another, or a combination of payment mechanisms from a third purchaser for a single service or set of services. This is what we refer to here as mixed provider payment systems (MPPS), which predominates in most countries [5]. Based on its particular design, each payment method creates specific incentives for provider behaviour. Yet, in combination, they may create a coherent or otherwise contradictory set of incentives for provider behaviour. In many countries, the different methods in place are not *aligned* with each other, producing conflicting incentives and thus influencing provider behaviour in an unconstructive way [6, 7]. This is revealed in detailed country studies on Mongolia, Vietnam, Morocco, and Burkina Faso, for example [8–11]. Provider behaviour is decisive insofar as it contributes to or impedes achieving health system objectives – that is, efficiency, equity in access, financial protection, and quality of care. Designing MPPS in a systematic way with aligned PPMs towards consistent incentives and effective provider behaviour is thus a critical part of efforts to enhance strategic purchasing [2, 7].

PPMs are considered a supply-side component of strategic purchasing policy. However, it is equally important to consider demand-side measures in purchasing, and in particular their *alignment* with provider payment. Demand- and supply-side measures interact and create expenditure uncertainty as well as incentives for providers, patients, and payers [4]. Demand-side measures include cost sharing, gatekeeping provisions, and referral rules as part of benefit package policies. How cost sharing aligns with existing PPMs is considered in this paper as a complementary strategy of provider payment. Evidence solely focusing on the effects of cost sharing mechanisms (without considering the interaction with supply-side measures) is mixed. Reviews of high-income countries determined that cost sharing for health care was not associated with a decrease in health expenditure and did not appear to significantly affect health care utilization or the distribution of out-of-pocket spending [12, 13]. On the other hand, drug cost sharing and reimbursement caps have been associated with reduced control of hypertension and hypercholesterolemia among US Medicare and Medicaid patients [14–16]. As such, alignment of cost sharing mechanisms embedded in MPPS becomes particularly important, as the degree of alignment may also influence the overall impact of the MPPS on health system objectives.

Past reviews on payment methods have generally focused in an isolated way on one individual payment method and its effects. However, the interplay of incentives within an MPPS and their influencing effects on provider behaviour, and ultimately on health system objectives, has so far not been adequately recognized nor analysed. The set of PPMs that comprise an MPPS are pieces of a complex adaptive system, in which health is jointly produced by services spanning various types of providers within the health system. Shifting to such a conceptualization of MPPS can support the optimization of coherent incentives for stakeholders across the system [2].

The objective of this paper is to review and synthesize the existing evidence on how *purposively* aligned MPPS affect efficiency and specifically health expenditure growth, equity in access to services, and quality. As part of the latter, particular focus is placed on coordinated and/or integrated care.

## Methods

### Conceptual framework

**Fig. 1:**
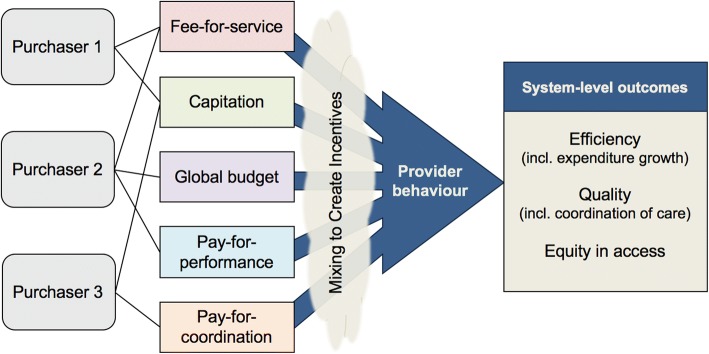
Conceptual framework of a mixed provider payment system with interacting incentives influencing provider behaviour toward health system-level outcomes

Figure 1 illustrates a representation of the conceptual framework linking purchasers to health system outcomes via a mix of provider payment methods. A diverse set of purchasers and combination of payment methods that they employ result in a set of incentives influencing provider behaviours that ultimately affect health system objectives. Examining reforms in which the payment methods are explicitly arranged to account for their interaction and to align incentives toward system objectives is key to optimizing policies for effective and efficient provider payment systems.

### Types of purposively aligned provider payment mixes

The predominant PPMs that are in place globally include salaries, FFS, payment per case or diagnosis-related groups (DRGs), capitation, line-item budget allocations, global budget, and pay-for-performance (PFP) methods. For a synthesis of the evidence, purposively aligned PPM mixes identified in this review were categorized into: (i) blended payment models, (ii) bundled payment models, (iii) cost-containment rewards typically added on top of a base payment, and (iv) cost sharing mechanisms aligned with the respective PPMs. Table 1 provides an overview of these categories with possible effects on provider behaviour. Country examples of these PPM mixes are presented in the Additional file 1.

**Table 1:**
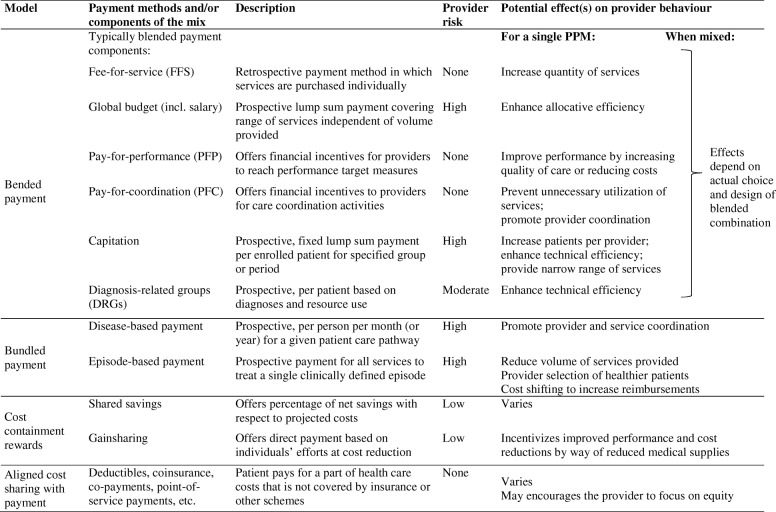
Overview of aligned provider payment mixes selected for this review

Source: Adapted from Charlesworth et al. (2012) [66] and OECD (2016) [17].

#### Blended payment

Blended payment models are characterized by a layering of individual PPMs (e.g. FFS, capitation, DRGs) and/or ‘add-on’ (e.g. PFP) incentives that are applied to individual or multiple providers [17]. For example, a blended payment arrangement could employ FFS plus (partial) capitation or FFS plus PFP. Pay-for-coordination (PFC) arrangements aim to promote the integration and coordination of care, improve efficiency, resource allocation, and funding [18]. Such arrangements that explicitly align the base payment with the add-on payment (PFP or PFC) have largely been designed in the context of Disease Management Programs (DMPs) for chronic care, as in Austria, France, and Germany.

#### Bundled payment

The term “bundling” refers to the degree to which components of health care are paid for together or separately [17]. Conventional forms of bundled payment include *capitation, case payment* or *diagnosis-related groups (DRGs)*, but these will not be further discussed here. We focus on payment models for service packages across different levels of care or across different specialties (e.g., pre- and post-hospitalization care, hospitalization, or chronic care). Bundled payment schemes are designed based on the expected costs of patient cases, episodes, or care over a specified time period and may be adjusted for the characteristics of a particular patient [7].

*Disease-based (or patient-based) bundled payment* remunerates providers on a per person (or enrolled member) per month (or year) (PMPM) level *for a given patient care pathway*, e.g., for type 2 diabetes or ischemic heart disease. Bundled care may include check-ups, specialist appointments, and related diagnostic tests for a chronic condition for as long as a year. A prospectively-determined amount covers a set of services based on historical costs, best practices, or clinical guidelines [7].

*Episode-based bundled payment* refers to a model in which a single payment to providers or facilities is paid for all services to treat a single episode of care [19, 20]. The fixed amount paid to the provider is calculated based on expected average costs for clinically defined episodes that may involve *several practitioner types, several settings of care, and several services or procedures over time*. Models surveyed in this review focused on bundles that include services beyond acute care, e.g. post-acute services, rehabilitation, and hospice care [21].^1^

#### Cost containment rewards

Shared savings and gainsharing arrangements may be included as additional components of blended or bundled payment schemes, and can explicitly promote the integration of care for a specific condition or across health needs. These specific arrangements may be made between the purchaser and provider to share cost savings or gains achieved through collaborative efforts involving shared risk.

*Shared savings* payment strategies offer providers a percentage of net savings with respect to projected costs as incentives to reduce health care spending for a defined patient population [22]. Shared savings agreements reward providers for using lowest-cost services to achieve desired outcomes. Moreover, through decreasing service utilization, these programs decrease revenues from direct patient payments to providers, for which providers must compensate through cost savings. This payment arrangement requires population health analytics, so that incentives across different care pathways can be adjusted based on patient outcomes.

*Gainsharing* arrangements involve direct payments to individual health employees based on cost reductions through their efforts and improved performance for specific sets of services [23]. Gainsharing can exist between a purchaser and individual physicians or between hospitals and physicians. The arrangement aims to promote savings as a result of quality improvements and increases in efficiency, rather than by decreases in utilization of high-cost services or increases in patients or productivity. This strategy is typically applied to specialties with high cost, high volume expenses, such as cardiovascular surgery, orthopaedic surgery, neurology, and oncology.

#### Aligned cost sharing

Cost sharing, whereby a patient pays for a part of health care costs that is not covered by health insurance or other schemes, is a critical demand-side mechanism. It includes deductibles, coinsurance, or co-payments associated with health insurance plans. Studies have identified positive and negative consequences of the implementation of cost sharing mechanisms for health financing. Out-of-pocket payment for health care may limit unnecessary use of health services, but may also impose barriers to seek care among the sick [13]. The incentives of cost sharing mechanisms operate within the context of the other incentives set through PPMs that providers face. The interplay of cost sharing and provider payment incentives compel the consideration of cost sharing mechanisms as components of MPPS and should be considered carefully for optimal design.

### Search strategy and literature review

A structured literature search was conducted in PubMed and databases of international agencies, including the World Health Organisation, the World Bank, and Organisation for Economic Cooperation and Development. Search terms related to mixed provider payment (e.g. strategic purchasing, active purchasing, blended payment, bundled payment, value-based purchasing), cost sharing, benefit design (e.g. benefit package design, referral rules), integrated care delivery and/or management, chronic disease management with financial incentives, demand-supply alignment, and cost shifting with multiple payers (see Table 2 for a full list of search terms).

**Table 2:** Search terms by category

Domain	Search terms
1. Purchasing	“strategic purchasing”
	“active purchasing”
2. Provider payment	“provider payment”
	“blended payment”
	“bundled payment”
	“value-based purchasing”
	“results based financing”
	“pay for performance”
3. Cost sharing	“cost sharing”
	“benefit package design”
	“benefit design”
	“referral rules”
4. Demand-supply alignment	demand
	supply
	alignment
	realignment
	“multiple payers”
5. Chronic conditions	“noncommunicable disease”
	“non-communicable disease”
	“chronic disease”
	“chronic-disease”
	“disease management”
6. Primary health care	“primary health care”
	“primary care”
	“primary prevention”
7. Integrated care	“integrated care delivery”
	“integrated care management”
	“care integration”
	“integrated care”
	“continuum of care”
	“provider network”
	“systems integration”
	“delivery of health care, integrated”
	“health delivery” AND “integration”

Note: Search terms within domains were combined using “OR”, and domains 1–4 were combined with other domains using “AND”

Articles primarily focused on multiple provider payment schemes and/or strategic purchasing were eligible for inclusion in this review. Titles and abstracts were screened according to these eligibility criteria. Based on this process, full-texts were obtained for further screening and data extraction. Articles focusing on effects of MPPS, strategic purchasing, and/or aligned cost sharing mechanisms on the objectives of (i) managing health expenditure growth, (ii) efficiency, and (iii) equity in access to care were included.

Given the focus on *alignment* of payment methods across multiple providers or services or interactions with the health system, the search placed particular emphasis on chronic and/or integrated or coordinated care. Articles that discussed individual PPMs only were not included. Only articles available in English were included. Commentaries, editorials, and opinion pieces that did not report on empirical evidence or experiences were also excluded. The final search was completed in December 2017. A PRISMA flow diagram is provided in the Additional file 2.

For selected articles, data on study objective, design, setting, payer(s), payment method, population covered, services covered, and effects on health expenditure growth, efficiency, quality of care, access to care, and integration of care were extracted to a Microsoft Excel spreadsheet. Studies were categorized by type (i.e. observed/empirical studies and/or modelling studies) and by payment model. Study findings on the effects on health expenditure growth, efficiency, and equity were qualitatively synthesized within these categories. Indications of health expenditure growth were often reported over time or as cost savings. Reported efficiency measures included length of stay, admission and readmission rates, discharge to home care versus rehabilitation facilities, and utilization of unnecessary services. Equity was assessed based on quality of care received and changes in access to care, particularly for traditionally marginalized groups of the study setting.

## Results

A total of 37 articles were included in this review, presenting findings on the effects of payment systems on managing health expenditure growth, increasing efficiency, or ensuring equity.

The majority of reviewed articles focused on high-income with 74% from the US; only three articles considered payment systems in upper-middle-income countries [24–26]. None of the articles discuss or report on experimental evidence comparing specific compositions of MPPS and/or alignment of cost sharing practices across a country’s system or by a single payer. Twelve blended payment models were reviewed as well as four examinations of adding PFP and three studies of adding a PFC element. Another 25 studies looked at bundled payment models. Five articles explored cost containment reward mechanisms (see Table 3).

**Table 3:** Summary of reviewed articles reporting effects on health expenditure growth, efficiency, and equity

Article					Effects			Author
Country	Year	Type of provider payment mix	Methods	Data	HE growth	Efficiency	Equity	
Australia	2015	Cost sharing (rebates)	Qualitative analysis	In-depth patient interviews			+	Foster & Mitchell [57]
		FFS-PFC blended payment					+	
Austria, Denmark, France, Germany	2016	FFS-PFC blended payment	Difference-in-differences analysis	Panel cost data	0			Tsiachristas et al. [27]
Austria, Germany	2012	FFS-PFC blended payment	Case study	Published literature, DISMEVAL project	+	+ / 0		Nolte et al. [47]
Belgium, Italy, Japan, Netherlands, Sweden, Taiwan, United Kingdom, United States	2012	Bundled payment	Systematic review	Published literature	+ (hospital, ambulatory)- (outpatient, post-acute)	+		Hussey et al. [19]
Canada	2011	CAP-PFP blended payment	Policy analysis	Published literature, semi-structured interviews with observers		+	+	Hutchison et al. [31]
		FFS-PFP blended payment				+		
Canada	2015	FFS-CAP blended payment	Nonlinear regression model	Population-based administrative records			+	Kiran et al. [33]
Canada	2015	FFS-PFP blended payment	Cost analysis with propensity score matching	Administrative records of costs and utilization by disease group	+ / -	+		Hollander & Kadlec [28]
China	2010	FFS-CAP blended payment	Systematic review	Published literature, official documents	0			Yip et al. [26]
		Pay-for-performance			+			
Estonia, Portugal, United Kingdom	2016	CAP-PFP blended payment	Difference-in-differences analysis	Panel cost data	+ (administrative, hospital)			Tsiachristas et al. [27]
France	2016	FFS-PFP blended payment	Difference-in-differences analysis	Panel cost data	+ (administrative, hospital)			Tsiachristas et al. [27]
Germany	2010	Pay-for-coordination	Multivariate regression analysis	Cohort study of type 2 diabetes patients		+		Schafer et al. [67]
Germany	2010	Pay-for-coordination	Cost analysis with propensity score matching	Insurance claims records	+	+		Stock et al. [68]
Germany, Netherlands	2016	Bundled payment	Difference-in-differences analysis	Panel cost data	+ (outpatient)			Tsiachristas et al. [27]
Hungary		CAP-PFC blended payment			0			
Netherlands	2012	Disease-based bundled payment	Case study	Published literature, DISMEVAL project	0			Nolte et al. [47]
Netherlands	2012	Disease-based bundled payment	Multilevel, random effects meta-analysis model	Individual patient data on performance indicators of processes and outcomes, DISMEVAL project		+		Elissen et al. [69]
Netherlands	2013	Disease-based bundled payment	Case study	Published literature, official documents	+	+		Froimson et al. [39]
Netherlands	2013	Disease-based bundled payment	Qualitative analysis	Semi-structured interviews with providers		+ / -		Raaijmakers et al. [70]
Netherlands, Germany	2014	Disease-based bundled payment	Case study	Published literature, expert interviews	–			Busse & Stahl [56]
		Shared savings			+			
Thailand	2015	FFS-CAP blended payment	Document review	Official and grey documents, published literature	+	+	+	Tangcharoensathien et al. [24]
United Kingdom	2009	Pay-for-performance	Multivariate logistic regression analysis	Cross-sectional surveys		+	+	Millett et al. [71]
United States	1995	Episode-based bundled payment	Case study	Narrative	+			Edmonds & Hallman [36]
United States	2007	Pay-for-performance	Descriptive analysis	Aggregated patient data	+	+		Casale et al. [72]
United States	2012	Pay-for-coordination	Systematic review	Peer-reviewed studies, published reports	+			Basu et al. [73]
United States	2013	Episode-based bundled payment	Case study	Document review	0			Chambers et al. [45]
United States	2014	Bundled payment (varied)	Issue brief	Document review	+	+		Bachrach et al. [34]
United States	2014	Episode-based bundled payment	Budget impact model	Cost data from the US Renal Data System	+			Liu et al. [42]
United States	2015	Episode-based bundled payment	Case study	Patient episode data	+	+		Doran & Zabinski [37]
United States	2015	Episode-based bundled payment	Experimental comparison study	Claims data	+	+		Froemke et al. [38]
United States	2015	Episode-based bundled payment	Case study	Claims data		+		Iorio [52]
United States	2015	Episode-based bundled payment	Comparative descriptive analysis	Acute care hospital participant data	0			Tsai et al. [48]
United States	2015	Episode-based bundled payment	Descriptive analysis	Patient and claims data, routine quality metrics	+ / 0	+		Whitcomb et al. [49]
United States	2015	Gainsharing	Experimental comparison study	Claims data	+	+		Froemke et al. [38]
United States	2015	Episode-based bundled payment	Case study	Narrative	+	+		Wagner [44]
		Pay-for-performance			+	+		
		Shared savings			+	+		
United States	2015	Shared savings	Case study	Narrative	+	+		Kuhn & Lehn [55]
United States	2016	Episode-based bundled payment	Descriptive analysis	Patient episode data	+	+		Bolz & Iorio [35]
United States	2016	Episode-based bundled payment	Experimental comparison study	Individual patient and episode reimbursement data	0	+		Courtney et al. [46]
United States	2016	Episode-based bundled payment	Case study	Narrative		+		Curry & Fee [50]
United States	2016	Episode-based bundled payment	Descriptive analysis	Medicare patient data	+	+		Iorio et al. [40]
United States	2016	Episode-based bundled payment	Cohort cost identification study	Insurance and commercial claims data	+ / 0			Kirby et al. [41]
United States	2016	Episode-based bundled payment	Issue brief	Narrative	+	+		Porter & Kaplan [43]
United States	2016	Episode-based bundled payment	Decision model with sensitivity analysis	Bundled payment claims data for patients discharged to rehabilitation and home		+		Slover et al. [53]
United States	2016	Patient-based bundled payment	Conceptual framework development synthesizing experiences from 6 cases	Published literature, official documents		+		Conrad et al. [51]
		Shared savings				+		

Note: (+) indicates improvements in indicator; (−) indicates worsening of indicator; (−/+) indicates mixed results; (0) indicates no changes and/or unclear findings; (+/0) indicates improvements reported, but with uncertain attribution to payment model of interest; empty indicates that this aspect was not studied. CAP = capitation; DRGs = diagnosis-related groups; EHR = electronic health record; FFS = fee-for-service; HE = health expenditure; PFC = pay-for-coordination; PFP = pay-for-performance

### Blended payment models

#### Effects on managing health expenditure growth

Four articles on blended models report moderate to no substantive reduction in expenditure growth compared to their previous non-blended payment model, but these results may be highly dependent on disease conditions and study follow up period [24, 26–28]. A review of strategic purchasing in Thailand found that implementation of capitation for outpatient services alongside fee schedules for selected conditions or services resulted in expenditure reductions compared to FFS only models [24]. A review of pilot reforms in China describes a set of reforms combining FFS payment with a disease-specific expenditure cap for each admission that indicates only moderate reduction in expenditure or no effect of this payment mix in comparison to the pre- FFS only model [26, 29]. While expenditures for diseases included in the payment model remained unchanged following reform implementation, costs for other diseases significantly increased, suggesting that unintended cost shifting occurred [26]. When PFP was added to an existing FFS system in British Columbia, Canada, effects on costs depended on the specific disease included in the scheme; the blended scheme resulted in cost savings for the management of hypertension, chronic obstructive pulmonary disease (COPD), and congestive heart failure, but not for diabetes management [28]. Authors attribute this difference to the exceptionally high costs of incentives for diabetes management.

A difference-in-differences (DID) analysis conducted using 1996 to 2013 panel data from 25 European countries assessed the impacts of payment reforms as *systems* [27]. This DID analysis determined that the introduction of PFC elements to FFS or capitation payment schemes decreased the growth of outpatient, hospital, medication, and administrative expenditures compared to pre-reform scenarios, but failed to have significant impact on the growth trajectories of total health expenditure. Those countries implementing PFP reforms, i.e. adding a PFP element to the existing payments, saw slight decreases in only hospital and administrative expenditure growth, but successfully reduced total expenditure growth. Observed changes in effects over time suggest that studies captured the immediate effects of reforms and that other long-term effects, such as those on total and medication expenditure growth, may not be apparent in the short-term.

#### Effects on efficiency

The few studies assessing efficiency focused on various Canadian payment reforms and reported increases in efficiency. Blending capitation for a basket of services with incentives for preventive services resulted in lower six-month prevalence of emergency department use compared to FFS-based blended models and simple FFS payment [30, 31]. The blending of FFS and performance payments resulted in primary care physicians providing more services, seeing more patients, making fewer referrals, and treating more complex patients compared to conventional FFS-only payment [31, 32]. The model included payment incentives (as a percentage of the FFS fee) to improve patient access and quality of care, such as premiums for extended hours, bonuses for chronic disease management, and incentives for patient enrolment into the program [32]. Incentives included bonus payments for comprehensive care services, including preventive services (e.g. pap smears, mammograms, childhood immunizations, flu shots, colorectal screening, annual health exam), a set of selected services (e.g. obstetrical deliveries, hospital services, palliative care, prenatal care, home visits), and chronic disease management (e.g. for diabetes, congestive heart failure, HIV) [32]. However, authors do not specify which specific services increased. Still, findings are suggestive of increased physician productivity as a result of this blended payment model [32].

Performance payments on top of usual FFS payments aiming to increase the provision of guidelines-based care to patients with chronic conditions (i.e., diabetes, congestive heart failure, COPD, and hypertension) resulted in fewer admissions, fewer days in hospital, fewer readmissions, and shorter lengths of stay across conditions [28]. These findings suggest that FFS blended with performance payments can reduce the need for costlier hospital services for multiple conditions. This early evidence points towards increases in efficiency as a result of blended payment models, particularly those blended with targeted performance payments. Additional research would add to the robustness of findings.

#### Effects on equity in access and receiving services

A study conducted in Ontario, Canada reports on the potential for blended payment models to promote recommended screening services in medical homes [33]. Diabetes patients enrolled in schemes in which 70% of providers’ earnings were based on capitation (and remaining 20% FFS and 10% other bonuses) were more likely to receive recommended testing compared to those in schemes with 15% capitation (and remaining 80% FFS and 5% other bonuses) [33]. While these results do not directly address equity concerns, they are suggestive of the potential for such blended methods to promote improved screening practices for populations that need them.

### Bundled payment models

#### Effects on managing health expenditure growth

Studies of bundled payment methods often reported reductions in expenditure compared to paying separately for the various service components, though with wide variation in magnitude. Thirteen articles in this review reported reductions in health expenditures associated with bundled payment reforms [19, 27, 34–44]. Four articles reported no or unclear effects of bundled payments on system-wide costs savings [41, 45–47].

Transition from an FFS reimbursement to bundled payment was generally associated with a decline in spending of up to 10% across eight high-income countries (Belgium, United Kingdom, Italy, Sweden, Taiwan, Japan, the Netherlands, and the United States) [19]. Likewise, the DID analysis conducted using 1996 to 2013 panel data from 25 European countries found that introduction of bundled payment schemes, rather than paying separately for individual service components via various other methods, reduced growth in outpatient and hospital expenditures, though failed to reduce total health expenditure growth [27]. Fifteen studies, however, were based in the United States, limiting most of the evidence to a distinct experience of provider payment and incentive structures [19, 34–43, 45–48]. Nine of these US-based articles examined bundled payment for joint replacement, further narrowing the scope of findings [34, 35, 37–40, 43, 46, 49]. The remaining articles also reported on specific diseases, such as end-stage renal disease and chronic heart failure. Cost reductions ranged from 8% to more than 30% across cases examining bundled payment for joint replacement in the United States [34, 35, 37–40, 43, 49].

Bundled payment holds promise for reducing expenditure growth in suitable cases based on context and managing specific conditions, but must be considered carefully due to wide variation in results. Reductions in health expenditure growth seem to depend significantly on benefit design, specific contracts, and the nature of disease management and related services. This suggests that research must ascertain optimal coverage of services under the specific payment models for each chronic condition of interest.

#### Effects on efficiency

Evaluations of bundled payment models found consistent increases in efficiency compared to implementing separate payment methods for individual services. Fifteen studies reported effects on efficiency, describing significantly shorter length of stay, decreased readmission rates, increases in discharge to home self-care, and reductions in utilization of services included in the bundle [19, 34, 35, 37–40, 43, 46, 47, 49–54]. Effects were associated with cost savings. A systematic review of the effects of bundled payment on health care in eight countries found that bundled payment was associated with 5 to 15% reductions in utilization of services included in the bundle [19]. However, the bulk of evidence was again limited to the experience of joint replacement in the United States. In the US, bundled payment for joint replacement consistently increased patient volume, decreased length of stay, decreased admission rates, and decreased discharge to inpatient rehabilitation facilities [35, 37–40, 43, 46, 49, 50, 52]. Based on the consistent findings associating bundled payment and increases in efficiency in the United States, similar effects may result for episode-based bundled payment for conditions requiring episode-based care as well as stages of long-term rehabilitation. These results warrant further research into bundled payment models and how they are designed and implemented to increase efficiency in care for specific conditions.

#### Effects on equity in access and receiving services

One article indirectly touched on equity concerns in the context of bundled payment for the management of solar keratosis, a precancerous skin lesion [41]. Study findings suggested that those not covered by the coverage scheme employing this payment model will have differential access to services and that this access will depend on design elements of the payment mix reforms, particularly the capacity of a purchaser to appropriately adjust for risk.

### Cost-containment rewards

Four articles focused on *shared* savings agreements were included in this review [44, 51, 55, 56], but only the last study referenced here discussed the impact of those additional agreements on health expenditure growth containment. Cost savings, often used as an indicator of efficiency, may point to potential reductions in health expenditure growth as well. Examples of shared savings and gainsharing arrangements in Germany and the United States, respectively, demonstrate that compared to non-blended payment models, the addition of such payment arrangements to other payment models may result in cost savings due to reduced inpatient length of stay, readmissions, or adverse events and complications. Specifically regarding health expenditure growth containment, the Healthy Kinzigtal (HK) initiative in Germany reported savings of US$203 per person per year in the enrolled population compared to the non-enrolled population in the first three years after its start [56]. These savings can be attributed to its integrated care model alongside a shared savings arrangement as an incentive to manage health expenditures.

In the United States, a bundled payment pilot for elective total joint replacement incorporated an optional *gainsharing arrangement* between the hospital and physicians [38]. Comparing pre- and post-pilot cohorts demonstrated a total savings of US$256,800, as a result of 63% of cases coming in at or under the previously negotiated price target [38]. Findings suggest that added incentives for coordination to generate cost savings that are shared with providers can contribute to effective management of health expenditure growth. However, it is unclear to what extent shared savings or gainsharing arrangements contribute to actual cost savings. It is possible that the bulk of savings result from the primary structure of the payment scheme, i.e., the total capitation model.

Overall, studies assessing efficiency due to shared savings and gainsharing arrangements are limited both in number and scope. A hospital system in the United States introduced a shared savings contract with four payers and reported a 13.1% reduction in emergency department visits, a 9.4% reduction in admissions, and a 13.4% reduction in CT scans that have also translated to cost savings [44]. The evaluation of the HK scheme reported increased admissions, which also increases expenditure, but decreased length of hospital stay per admission (i.e., an efficiency increase) [56]. However, the extent to which these effects can be attributable to shared savings agreements versus the scheme’s overall efforts to integrate care remains unclear. Furthermore, it is questionable as to whether these impacts have positive effects on health outcomes. The reasons for the reductions in admissions and CT scans should particularly be examined to ensure that these are truly improvements in both efficiency and quality. Additional research should consider capturing the effects of these schemes surrounding positive gains in efficiency versus any increases in efficiency.

### Alignment of cost sharing mechanisms and payment methods

Only one study in this review considered the alignment of cost sharing mechanisms in relation to the payment of allied health professionals [57]. In this case, 13 allied health services in the primary care sector were paid directly by patients with publicly funded rebates available through Medicare to reduce or offset out-of-pocket expenditure [57]. The cost sharing rebates in the Australian case constitute an example of aligning cost sharing practices with payments methods to encourage greater coordination of care, better access to these services of secondary prevention, and potentially other objectives, such as equity. Increased referrals to allied health services have suggested positive impacts in access to care, particularly among lower socioeconomic groups [57, 58]. However, additional studies of this reform warn of ongoing concerns about equitable access to specific services for those with increasingly complex conditions that are not addressed by this cost sharing rebates program [57–60]. Moreover, existing research does not explore the impacts of the initiative in terms of managing health expenditure growth and increasing efficiency.

Given this limited evidence, it is difficult to support conclusive evidence on the impact of alignment of cost sharing mechanisms and provider payments on health expenditure growth. Similarly, there is a lack of evidence around how cost sharing practices in alignment with PPMs can be optimized to increase efficiency and work towards more equitable access to services for chronic disease.

## Discussion

This review adopted a conceptual framework that accounted for a mixed provider payment system with multiple payment methods, through which funds from multiple purchasers are channelled to multiple types of providers and examined its impact on health system-level outcomes, often designated goals of health financing reforms. Synthesis of relevant studies revealed containment of health expenditure growth under bundled payment models and cost containment rewards across the countries studied. Yet, studies from Hungary, the Netherlands, Germany, and the US reported initial increases in costs or unclear effect of reforms, suggesting that impacts on costs may be absent or delayed. Availability of evidence in this regard remains limited as there may simply be fewer published articles describing instances in which payment reform failed to appropriately manage expenditure growth. On the other hand, blended payment models, i.e., the combination of two or several payment methods for a defined service or set of services, generally reported moderate to no substantive reductions in expenditure growth.

Assessing effects of MPPS on efficiency was equally prominent across this review, with 26 articles examining efficiency effects, measured in terms of length of stay and utilization of specific services, such as emergency department visits and patient readmission. Nearly all articles across the various MPPS studies reviewed reported decreased length of stay and readmissions as well as increased discharge to home care. Often, a primary objective of payment system reforms is to improve efficiency, i.e., to reduce duplicative, unnecessary services towards more integrated care provision. In the face of an increasing burden of chronic disease around the world, better integration of health care systems (incl. Provider payment methods) for improved management of health expenditure, efficiency, quality of care, and health outcomes has been increasingly recognized.

The “Project INTEGRATE”, research supported by the European Commission, shows that fragmented financial systems or provider payment methods may create barriers to care integration [7]. Their findings support the idea that bundled payment can facilitate care integration, but also that such schemes may face challenges in appropriately adapting to patient needs. Their report also demonstrates that questions remain surrounding impacts on quality of care and ultimate health outcomes [7]. The framework proposed by Stokes et al. (2018) that aims to assess the level of integration fostered through various features of payments can guide future research on effective payment models and their impact on efficiency, quality and equity [61].

Our review further shows that evidence on equity in access to services is limited, though suggestive of positive impacts in studies from Australia, Canada, Thailand, and the UK. The objective of ensuring equity in access and receiving services does not appear to be a priority for evaluation in existing research studies, making it difficult to draw conclusions about the actual effect of MPPS on equity. Nevertheless, they are suggestive of the idea that MPPS may be able to promote the provision of specific services for populations that need them.

Existing studies are suggestive of the potential for greater alignment of demand and supply side incentives to achieve objectives, particularly management of health expenditure growth. However, studies also acknowledge the potential limits of cost sharing mechanisms as components of integrated or complex disease management programs. A cross-sectional study of the United States using 2007 Medical Expenditure Panel Survey data determined that moving to high cost sharing policies for physician care significantly reduced total health care expenditure more for chronically ill individuals than for healthy people [62]. Authors discuss that this greater expenditure reduction among the sick was attributable to decreased utilization and may have deprived patients of needed care, risking health outcomes [62]. A related study comparing individuals with and without chronic conditions shows that high cost sharing policies similarly affects the utilization of patients without chronic diseases [63]. Based on these findings, authors warn that greater cost sharing may result in higher need for costly medical care in the long run, ultimately resulting in overall higher expenditure [63]. Nevertheless, these point to the potential opportunities where greater incentive alignment could be better realized.

While this paper looked at the effects of MPPS, their impact is equally contingent upon institutional factors that allow provider networks and care coordination to function. The most important factor in this respect is the service delivery model itself, which needs to enable care coordination and enhance provider networks, thereby overcoming solo practice and optimising staff mix. Good governance and appropriate levels of purchaser and provider autonomy, support to all players, and cooperation among the major purchasers and provider organizations is critical [51, 64].

Specific gaps in evidence include well-designed experiments and robust analyses on where, when, and how MPPS and cost sharing practices align towards achieving goals across purchasers, providers, and patients. In particular, the equity dimension is missing from these analyses; identifying specific design elements for equity in accessing health care and receiving services will be critical to designing MPPS for UHC. Another key limitation of studying the effects of MPPS is that, in many cases, multiple health system and financing reforms occur simultaneously, making it difficult to fully attribute effects to any single payment reform. In fact, reviewers consistently rate evaluative studies of payment reform as being of low quality due to confounding and other factors. Moreover, these payment reforms have not been assessed with a conceptual framework and logic that focuses on the specifics of an MPPS. It is important to acknowledge that publication bias may play a role in the predominantly positive results seen in the existing literature; the scarcity of peer-reviewed experimental studies among the 37 articles on MPPS may be due to the limited to no effect of payment reforms.

Finally, the majority of examples outlined in this review come from the United States and other high-income settings, which, in view of their health system context and resource availability, may make lessons less relevant to other settings. Good design and implementation is contingent upon a number of institutional requirements, such that it may not be easy to replicate those experiences in low- and middle-income countries. Overall, aligned MPPS do not yet seem to be widespread, with the exception of layering a base payment with PFP. The blending of payment methods can be incrementally introduced. Such incremental processes may prove less complex in implementation than reform measures required to establish system-wide changes, which may be the case for bundled payment models. Individual PPMs in blended systems can also be adjusted over time as capacity of purchasers and providers increases and as information management systems improve.

## Conclusions

The existing evidence hints at the far-reaching effects of different MPPS across the health system potentially mediated by numerous factors to achieve objectives. This review also suggests that the effects of a particular MPPS are highly context-specific to a country or region and service line. Successful design and implementation will require adaptation and research based on population needs, expected challenges, and also depend on the resources available. Identifying where the potential for MPPS alignment exists will be a step towards rigorously examining their effects on key objectives. Measuring the impact of different MPPS on expenditure growth, efficiency, and equity of health systems means delineating to what extent impacts in these areas can be attributed to individual PPMs, aligned MPPS or cost sharing mechanisms.

Planners and policymakers should consider the existing system, specific goals of reform, and feasibility in realizing implementation when designing an MPPS. Stakeholder participation, ownership, and leadership in the adoption and implementation of payment reforms are equally important [65]. Across the transition, the strong commitment and participation of leadership is critical. Available information technology should be used to monitor and scale programs; in the case of value-based payment systems, information technology can be essential in achieving efficiency while identifying good performers. This points to the importance of perceiving and embedding alignment of payment methods and MPPS reforms as part of a broader transformation of the health system and specifically of the service delivery model.

In view of the limited existing evidence with focus on high-income countries and on the US, there is a need to build research endeavours around the idea that a single PPM functions as part of a larger MPPS. A guiding framework to determine how to study and evaluate MPPS in terms of managing health expenditure growth, increasing efficiency, and ensuring equity across contexts is warranted. Future research should consider a conceptual framework in which the complex nature of MPPS is explicitly acknowledged. Future evidence generation should focus on the effectiveness and implementation of MPPS reforms in a greater diversity of settings.

## Additional files

Additional file 1: Examples of mixes of provider payment methods. Summary of literature. Literature based examples of mixes of provider payment methods – 6 Boxes. (DOCX 239 kb)

Additional file 2: PRISMA flow chart. (DOCX 32 kb)

## Footnotes

1 Complete bundling turns into total capitation that is provided for the totality of care for a defined population (e.g. Accountable Care Organizations in the United States; Healthy Kinzigtal in Germany [21, 56]. Because it is beyond the focus of this paper on *aligned* mixed payment systems, this payment model is not reviewed here.
