# Supplementary material for: Effects of mixed provider payment systems and aligned cost sharing practices on expenditure growth management, efficiency, and equity: a structured review of the literature
Source: BMC Health Serv Res. 2018 Dec 27;18:996. doi: 10.1186/s12913-018-3779-1 (PMC6307240; doi:10.1186/s12913-018-3779-1)
Supplement: Supplementary file 1 — Examples of mixes of provider payment methods. Summary of literature. Literature based examples of mixes of provider payment methods – 6 Boxes. (DOCX 239 kb) [file 12913_2018_3779_MOESM1_ESM.docx]

**Appendix 1. Country examples of aligned provider payment mixes**

**Box 1. Pay-for-performance and fee-for-service payment for primary care in British Columbia, Canada**

Primary health care services and drugs are covered benefits for which providers bill provincial government insurance programs directly. The majority of medical services are billed on a fee-for-service basis. Due to a decline in the number of full-service family practitioners in the early 2000s, regional authorities have developed and implemented initiatives to promote enhanced family practice. The approach provided general practitioners with “add-on” fixed payments in the FFS system for “enhanced care”, including the development of care plans and taking more time for patients with chronic or complex conditions. Incentives were implemented for general practitioners to provide enhanced, guideline-based care to patients with diabetes and congestive heart failure, and expanded to other chronic conditions over time. [1,2]


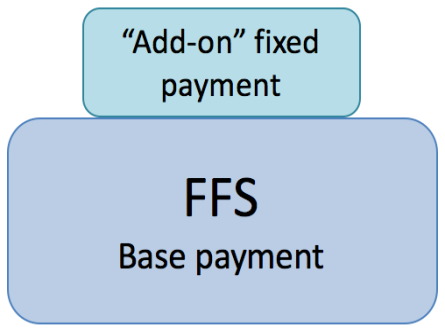


**Box 2. Pay-for-coordination in the Health Insurance Reform Act of 2004 in France**

The Health Insurance Reform Act of 2004 targeted primary care providers in France by promoting the expanded use of Disease Management Programs (DMPs) for 30 chronic conditions, including diabetes, chronic obstructive pulmonary disease (COPD), cardiovascular diseases, musculoskeletal diseases and certain cancers. The aim of the program was to improve quality of care, patient monitoring, promote continuous medical education of care guidelines, alleviate the financial burden associated with unnecessary procedures, and strengthen the role of the general practitioner. The law included a pay-for-coordination (PFC) scheme in which general practitioners received a supplemental 40 EUR per patient up to 6,000 EUR annually (30% of their base payment) for care coordination. [3,4]

**Box 3. Bundled payment for chronic conditions in the Netherlands**

Provider networks called “care groups” are paid a single fee for the full range of outpatient services for a specific chronic condition over a designated time period that is negotiated between insurers and care groups. This allows the different components of care for a chronic condition to be packaged as a single product. Care groups can deliver the various components of care or subcontract other types of providers across the health system to do so. The amount paid for the bundle of services is negotiated in advance between each care group and insurers. The fees for individual subcontractors are correspondingly freely negotiable between care groups and care professionals. The services included in the bundle follow national care standards, which is based on evidence-based guidelines for primary care physicians. To foster task redistribution, standards describes care in terms of functions rather than providers. [4,5,6,7,8,9-11]

**
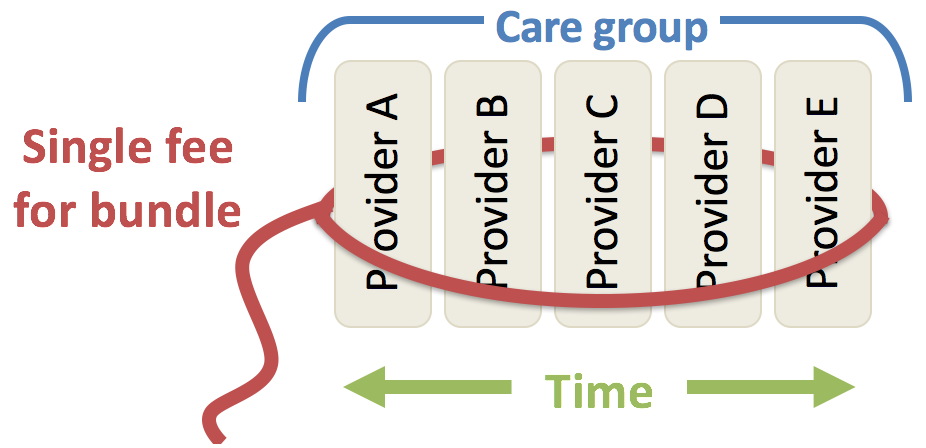
**

**Box 4. Episode-based bundled payment for joint replacement in the United States**

The Centers for Medicare & Medicaid Services (CMS) began the Bundled Payment for Care Improvement (BPCI) initiative in 2011 to align incentives among individual health care providers within the admitting hospital and reduce the cost to Medicare for joint replacements. The total episode of care covered by BPCI was defined as all costs associated with the initial hospital admission from the time of surgery and all care up to 90 days post-discharge. These services included inpatient admission, post-discharge care (e.g. skilled nursing facility, inpatient rehabilitation, or home care), outpatient care, laboratory and radiology charges, readmission, and durable medical equipment. CMS monitors the period from 91 to 120 days following an episode to ensure that services are not being shifted outside of the bundle. The provider organization is held financially responsible if such violations occur.

The initiative explicitly emphasized reducing length of stay in its design through early rehabilitation and standardized pain protocols. Patients enrolled in the initiative were flagged in the electronic medical record to alert providers when considering discharge or readmission to the hospital.

Financial compensation by CMS to the provider organization is through retrospective reconciliation of all the charges against the predetermined target price after the episode of care. All physicians involved in the operation and episode at the initiator hospital are included in the bundle and any patient having joint replacement surgery is by default part of the bundle to guard against exclusion of higher risk patients or populations. [12,13,14,15,16,17-20,21,22,23,24]

**Box 5. Shared savings payment by “Healthy Kinzigtal Integrated Care” in Germany**

“Healthy Kinzigtal” (HK) is a regional health management company comprised of 86 care providers, including general practitioners, specialists, hospitals, nursing homes, and ambulatory home health agencies as well as pharmacies, health and sports clubs, adult education centres, and local governments. The HK company organizes and provides care across all health service sectors for all ages and health care needs. Providers are reimbursed by sickness funds on the basis of the existing payment methods and rates in place. However, when the total costs of care for all insured affiliates in the Kinzigtal region are lower than the risk-adjusted standardized payments, i.e. average costs across all pools, the respective sickness fund and HK company share the savings. A proportion of these funds are allocated to financial incentives for providers. [25,26]


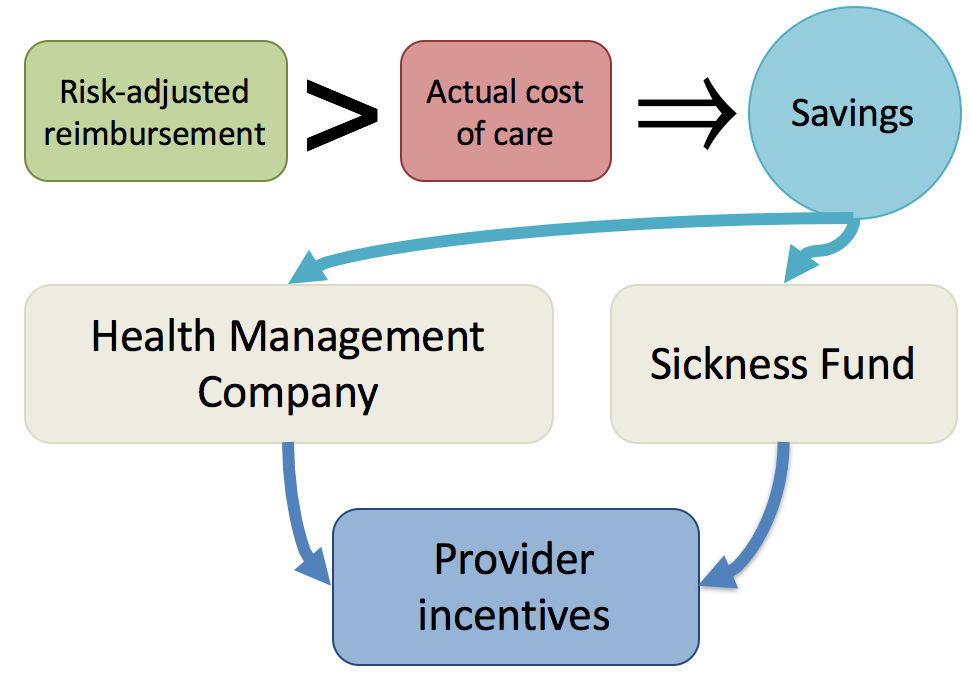


**Box 6. A pilot gainsharing model for joint replacement in the United States**

A bundled payment model that included a gainsharing arrangement for elective joint replacement was developed and implemented at two high volume hospitals in the United States. A fixed payment amount covered an episode of care beginning 30 days pre-operatively and ended 90 days post-discharge for all contracted services within this time frame. Cases that resolved below this payment price and met quality targets imply a saving and resulted in a payment that was allotted to a hospital/physician gainsharing pool. Physicians were eligible to gainshare when cases met quality group and individual targets, which had been determined by purchasers, hospitals, and physicians.

At the group level, two quality targets must be met for the savings pool to exist: (1) 95% of cases must have passed Surgical Care Improvement Project (SCIP) measures and (2) not more than 26% of cases were discharged to a skilled nursing facility. The expectation is that these measures motivate physicians to improve group practices. If these group targets are met, each physician is individually accountable for meeting four quality measures in order to earn a payout: (1) passed SCIP, (2) no mortality, (3) completed provider-administered questionnaire on patient condition, and (4) no related readmission. [18]

**References:**

1. Hollander MJ, Kadlec H. Incentive-Based Primary Care: Cost and Utilization Analysis. Perm. J. [Internet]. 2015;19:46–56. Available from: http://www.ncbi.nlm.nih.gov/pubmed/26263389
2. Hutchison B, Levesque J-F, Strumpf E, Coyle N. Primary health care in Canada: systems in motion. Milbank Q. [Internet]. 2011;89:256–88. Available from: http://www.ncbi.nlm.nih.gov/pubmed/21676023
3. Tsiachristas A, Dikkers C, Boland MRS, Rutten-van Mölken MPMH. Exploring payment schemes used to promote integrated chronic care in Europe. Health Policy [Internet]. 2013;113:296–304. Available from: http://www.ncbi.nlm.nih.gov/pubmed/23937868
4. Tsiachristas A, Dikkers C, Boland MRS, Rutten-van Mölken MPMH. Impact of financial agreements in European chronic care on health care expenditure growth. Health Policy [Internet]. 2016;120:420–30. Available from: http://www.ncbi.nlm.nih.gov/pubmed/26971018
5. Nolte E, Knai C, Hofmarcher M, Conklin A, Erler A, Elissen A, et al. Overcoming fragmentation in health care: chronic care in Austria, Germany and The Netherlands. Health Econ. Policy. Law [Internet]. 2012;7:125–46. Available from: http://www.ncbi.nlm.nih.gov/pubmed/22221931
6. Busse R, Stahl J. Integrated care experiences and outcomes in Germany, the Netherlands, and England. Health Aff. (Millwood). [Internet]. 2014;33:1549–58. Available from: http://www.ncbi.nlm.nih.gov/pubmed/25201659
7. Elissen AMJ, Duimel-Peeters IGP, Spreeuwenberg C, Spreeuwenberg M, Vrijhoef HJM. Toward tailored disease management for type 2 diabetes. Am. J. Manag. Care [Internet]. 2012;18:619–30. Available from: http://www.ncbi.nlm.nih.gov/pubmed/23145806
8. Raaijmakers LG, Hamers FJ, Martens MK, Bagchus C, de Vries NK, Kremers SP. Perceived facilitators and barriers in diabetes care: a qualitative study among health care professionals in the Netherlands. BMC Fam. Pract. [Internet]. 2013;14:114. Available from: http://www.ncbi.nlm.nih.gov/pubmed/23937325
9. de Bakker DH, Struijs JN, Baan CB, Raams J, de Wildt J-E, Vrijhoef HJM, et al. Early results from adoption of bundled payment for diabetes care in the Netherlands show improvement in care coordination. Health Aff. (Millwood). [Internet]. 2012;31:426–33. Available from: http://www.ncbi.nlm.nih.gov/pubmed/22323174
10. Busetto L, Luijkx K, Huizing A, Vrijhoef B. Implementation of integrated care for diabetes mellitus type 2 by two Dutch care groups: a case study. BMC Fam. Pract. [Internet]. 2015;16:105. Available from: http://www.ncbi.nlm.nih.gov/pubmed/26292703
11. de Bruin SR, van Oostrom SH, Drewes HW, de Jong-van Til JT, Baan CA, Struijs JN. Quality of diabetes care in Dutch care groups: no differences between diabetes patients with and without co-morbidity. Int. J. Integr. Care [Internet]. 2013;13:e057. Available from: http://www.ncbi.nlm.nih.gov/pubmed/24409109
12. Hussey PS, Mulcahy AW, Schnyer C, Schneider EC. Closing the quality gap: revisiting the state of the science (vol. 1: bundled payment: effects on health care spending and quality). Evid. Rep. Technol. Assess. (Full. Rep). [Internet]. 2012;1–155. Available from: http://www.ncbi.nlm.nih.gov/pubmed/24422914
13. Bolz NJ, Iorio R. Bundled Payments: Our Experience at an Academic Medical Center. J. Arthroplasty [Internet]. 2016;31:932–5. Available from: http://www.ncbi.nlm.nih.gov/pubmed/27020651
14. Slover JD, Mullaly KA, Payne A, Iorio R, Bosco J. What is the Best Strategy to Minimize After-Care Costs for Total Joint Arthroplasty in a Bundled Payment Environment? J. Arthroplasty [Internet]. 2016;31:2710–3. Available from: http://www.ncbi.nlm.nih.gov/pubmed/27344351
15. Ridgely MS, de Vries D, Bozic KJ, Hussey PS. Bundled payment fails to gain a foothold In California: the experience of the IHA bundled payment demonstration. Health Aff. (Millwood). [Internet]. 2014;33:1345–52. Available from: http://www.ncbi.nlm.nih.gov/pubmed/25092835
16. Hussey PS, Ridgely MS, Rosenthal MB. The PROMETHEUS bundled payment experiment: slow start shows problems in implementing new payment models. Health Aff. (Millwood). [Internet]. 2011;30:2116–24. Available from: http://www.ncbi.nlm.nih.gov/pubmed/22068404
17. Doran JP, Zabinski SJ. Bundled payment initiatives for Medicare and non-Medicare total joint arthroplasty patients at a community hospital: bundles in the real world. J. Arthroplasty [Internet]. 2015;30:353–5. Available from: http://www.ncbi.nlm.nih.gov/pubmed/25680450
18. Froemke CC, Wang L, DeHart ML, Williamson RK, Ko LM, Duwelius PJ. Standardizing Care and Improving Quality under a Bundled Payment Initiative for Total Joint Arthroplasty. J. Arthroplasty [Internet]. 2015;30:1676–82. Available from: http://www.ncbi.nlm.nih.gov/pubmed/26092251
19. Froimson MI, Rana A, White RE, Marshall A, Schutzer SF, Healy WL, et al. Bundled payments for care improvement initiative: the next evolution of payment formulations: AAHKS Bundled Payment Task Force. J. Arthroplasty [Internet]. 2013;28:157–65. Available from: http://www.ncbi.nlm.nih.gov/pubmed/24034511
20. Iorio R, Clair AJ, Inneh IA, Slover JD, Bosco JA, Zuckerman JD. Early Results of Medicare’s Bundled Payment Initiative for a 90-Day Total Joint Arthroplasty Episode of Care. J. Arthroplasty [Internet]. 2016;31:343–50. Available from: http://www.ncbi.nlm.nih.gov/pubmed/26427938
21. Porter ME, Kaplan RS. How to Pay for Health Care. Harv. Bus. Rev. [Internet]. 2016;94:88–98, 100, 134. Available from: http://www.ncbi.nlm.nih.gov/pubmed/27526565
22. Courtney PM, Ashley BS, Hume EL, Kamath AF. Are Bundled Payments a Viable Reimbursement Model for Revision Total Joint Arthroplasty? Clin. Orthop. Relat. Res. [Internet]. 2016;474:2714–21. Available from: http://www.ncbi.nlm.nih.gov/pubmed/27357691
23. Tsai TC, Joynt KE, Wild RC, Orav EJ, Jha AK. Medicare’s Bundled Payment initiative: most hospitals are focused on a few high-volume conditions. Health Aff. (Millwood). [Internet]. 2015;34:371–80. Available from: http://www.ncbi.nlm.nih.gov/pubmed/25732486
24. Whitcomb WF, Lagu T, Krushell RJ, Lehman AP, Greenbaum J, McGirr J, et al. Experience with Designing and Implementing a Bundled Payment Program for Total Hip Replacement. Jt. Comm. J. Qual. Patient Saf. [Internet]. 2015;41:406–13. Available from: http://www.ncbi.nlm.nih.gov/pubmed/26289235
25. Scott A, Tjosvold L, Chojecki D. Gainsharing and shared savings strategies in the healthcare setting: Evidence for effectiveness. Alberta, Canada; 2016 Nov.
26. Busse R, Stahl J. Integrated care experiences and outcomes in Germany, the Netherlands, and England. Health Aff. (Millwood). [Internet]. 2014;33:1549–58. Available from: http://www.ncbi.nlm.nih.gov/pubmed/25201659

**Appendix 2. PRISMA flow diagram of search results**

Records identified through

database searching
(n = 6,773)

Titles and abstracts screened
(n = 6,424)

Duplicate records removed
(n = 349)

Records excluded based on

inclusion / exclusion criteria
(n = 6,271)

Full-text articles assessed for eligibility

(n = 153)

Records excluded
(n = 116)

Primary reasons for exclusion:

- No empirical evidence presented
- No purposive alignment of payment methods

Studies included in synthesis
(n = 37)
